# Acute rhabdomyolysis with severe polymyositis following ipilimumab-nivolumab treatment in a cancer patient with elevated anti-striated muscle antibody

**DOI:** 10.1186/s40425-016-0139-8

**Published:** 2016-06-21

**Authors:** Mehmet Asim Bilen, Sumit K. Subudhi, Jianjun Gao, Nizar M. Tannir, Shi-Ming Tu, Padmanee Sharma

**Affiliations:** Division of Cancer Medicine, The University of Texas MD Anderson Cancer Center, Houston, TX USA; Department of Genitourinary Medical Oncology, The University of Texas MD Anderson Cancer Center, Unit 1374, 1155 Pressler Street, Houston, TX 77030-3721 USA

**Keywords:** Immune checkpoint inhibitors, Ipilimumab, Nivolumab, Rhabdomyolysis, Polymyositis, Immune-related adverse event, Urothelial carcinoma

## Abstract

**Background:**

Immune checkpoint inhibitors have revolutionized cancer therapy since these drugs target inhibitory pathways on T cells, which result in durable anti-tumor immune responses and significant overall survival for a subset of cancer patients. These drugs can also lead to toxicities, which require additional research to identify mechanisms of toxicities and biomarkers that can help to identify patients who will develop immune-related adverse events.

**Case presentation:**

We describe the first case, to our knowledge, of a patient with metastatic urothelial carcinoma who developed acute rhabdomyolysis with severe polymyositis after treatment with combination immunotherapy consisting of ipilimumab plus nivolumab (Trial registration: NCT01928394. Registered: 8/21/2013). We found that this patient had an elevated pre-existing anti-striated muscle antibody titer, which was likely exacerbated with the immunotherapy treatment thereby resulting in the presentation of acute rhabdomyolysis and severe polymyositis.

**Conclusions:**

This case suggests that immune-related adverse events may be linked to subclinical autoimmune conditions which highlights the need for additional studies to identify patients who are at risk for toxicities.

## Background

Advances in our understanding of immune checkpoints have led to the development of novel approaches for cancer therapy. Ipilimumab is a monoclonal antibody that directly inhibits the function of the immune checkpoint cytotoxic T-lymphocyte antigen 4 and it was shown to improve survival, which led to approval in treatment of patients with melanoma [1, 2]. Nivolumab targets the programmed death-1 checkpoint and was approved as treatment for patients with metastatic melanoma, non-small cell lung cancer and renal cell carcinoma [3–5]. Recently, combination therapy with nivolumab plus ipilimumab was shown to improve progression free survival, which led to approval of this combination as treatment for patients with metastatic melanoma [6].

Although immune checkpoint therapies can lead to significant clinical benefit, they are also associated with immune-related adverse events (irAEs). As increasing numbers of these immunomodulatory treatments are under investigation, clinicians need to become familiar with diagnosing and controlling irAEs. More importantly, immunologic mechanisms and biomarkers that can help to identify patients at risk for irAEs with immune checkpoint therapy are critical to help clinicians select appropriate patients for treatment. We describe here the first case, to our knowledge, of a patient with metastatic papillary transitional cell carcinoma after three prior chemotherapy regimens, in whom acute rhabdomyolysis with severe polymyositis developed on a clinical trial of ipilimumab plus nivolumab. We retrospectively identified an elevated pre-existing anti-striated muscle antibody titer (anti-SM-titer), which provides a potential immunologic mechanism and biomarker for patients at risk for rhabdomyolysis and polymyositis with immune checkpoint therapy.

## Case presentation

The patient, a 73-year-old Chinese man, with no significant past medical history, presented with hematuria and was initially treated with a left nephroureterectomy in May 2009. The pathology report showed a papillary transitional cell carcinoma in the renal pelvis measuring 2.6 cm with evidence of lymphovascular invasion. At that time, the patient did not receive adjuvant chemotherapy and did well until October 2010.

In October 2010, the patient underwent staging workup which revealed multiple pulmonary metastases. He received 7 cycles of gemcitabine combined with carboplatin. Initially, he experienced response to treatment. However, there were residual pulmonary nodules and these were noted to be stable in repeat restaging study.

In September 2011, the patient sought evaluation at the University of Texas MD Anderson Cancer Center for further care. Due to residual pulmonary nodules, he received 2 courses of dose-dense MVAC (Methotrexate 30 mg/m2, doxorubicin 30 mg/m2, vinblastine 3 mg/m2, cisplatin 70 mg/m2) without further improvement of the residual lung nodules. After this, he was followed closely without further treatment.

In July 2013, he presented with dysuria. He underwent cystoscopy which confirmed tumor involvement in the bladder. In August 2013, he underwent a radical cystoprostatectomy. Pathology revealed a stage 4a urothelial carcinoma with invasion into the prostatic stroma, surgical margins were negative, and none of the lymph nodes were involved by metastatic disease. He received 3 cycles of chemotherapy consisting of dose-dense MVAC, which was completed in November 2013. After this, he was followed closely without further treatment.

In August 2014, he presented with progressive metastatic disease including new right external iliac lymphadenopathy and increase in size of pulmonary nodules. He received 3 cycles of pemetrexed every 3 weeks (500 mg/m2) with no detectable change in the size of measurable disease.

In November 2014, the patient agreed to participate in a randomized phase 1/2, open-label study of nivolumab monotherapy or nivolumab combined with ipilimumab (approved by the Institutional Review Board). He was randomly assigned to receive treatment with nivolumab (3 mg/kg) combined with ipilimumab (1 mg/kg) every 3 weeks. He received the first treatment on November 14, 2014, which was well-tolerated with no immediate adverse effects. On December 4, 2014, he received his second treatment. At that time, he reported mild throat discomfort and tightness in his jaw when he swallowed; the etiology of these symptoms was unclear and not thought to be related to the immunotherapy agents. The patient denied any skin rash, diarrhea, or abdominal pain. His blood test results were within normal limits, except for an asymptomatic alanine transaminase (ALT) level of 157 IU/L and an aspartate transaminase level (AST) of 703 IU/L, which was a grade 3 adverse event per the Common Terminology Criteria for Adverse Events, Version 4 (CTCAE). Since the patient did not have any clinical symptoms such as abdominal pain or nausea/vomiting associated with elevated liver function test (LFTs), a plan of close observation without changes in treatment was instituted as per the guideline of clinical trial protocol.

On December 8, 2014, the patient presented to the emergency department with complaints of significant lower back pain, profound weakness including inability to ambulate, and difficulty opening his mouth, eating, and speaking. On physical examination, the patient was noted to have bilateral ptosis and extraocular muscle weakness. His vital signs were stable. Laboratory tests showed creatine kinase (CK) level of 13,710 U/L (reference range; 55–170, CTCAE grade 4), AST level of 1346 IU/L (reference range; 15–46, CTCAE grade 4), ALT level of 378 IU/L (reference range;7–56, CTCAE grade 3), CK-MB level of 149.1 ng/mL (reference range; 0.6–6.3), creatinine level of 1.5 mg/dL (reference range; 0.7–1.3), blood urea nitrogen level of 43 mg/dL, troponin I level of 7.98 ng/mL (reference range; <0.03), alkaline phosphatase level of 44 U/L (reference range; 38–126), lactate dehydrogenase level of 3697 IU/L (reference range; 313–618), and the presence of myoglobinuria. An electrocardiogram was unremarkable. The patient was admitted to the intensive care unit for further care and subsequently diagnosed with acute rhabdomyolysis associated with severe polymyositis, which were suspected to be immune-related toxicities. He received intravenous fluids and methylprednisolone (1 mg/kg twice daily).

**Fig. 1:**
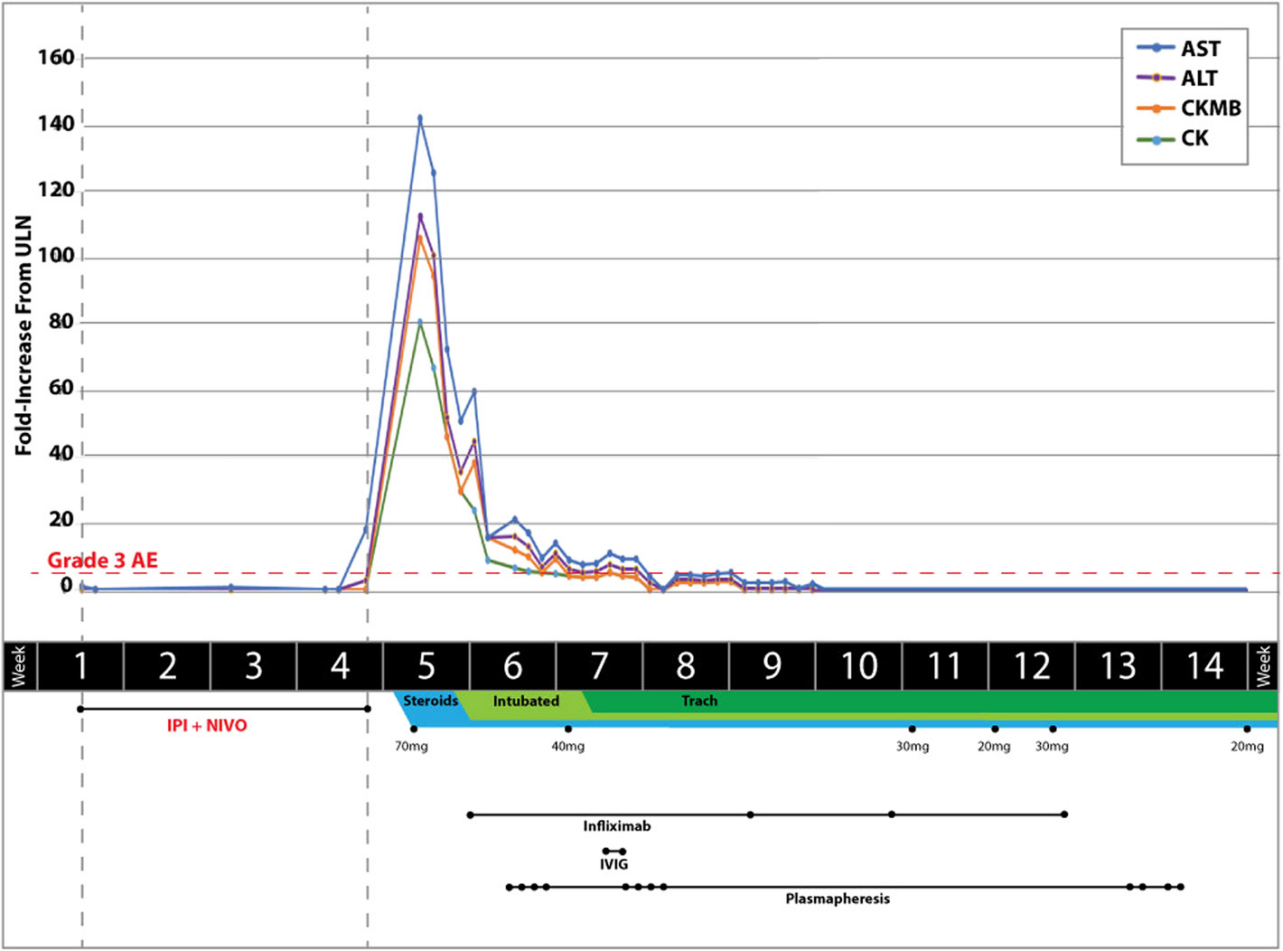
Summary of the patient’s hospital stay including changes in laboratory results and treatment. Dotted lines indicate grade 3 adverse events according to the Common Terminology Criteria for Adverse Events, Version 4. AST indicates aspartate transaminase; ALT, alanine transaminase; CK-MB, creatinine kinase isoenzyme MB; CK, creatinine kinase; ULN, upper limit of normal; AE, adverse event; IPI, ipilimumab; NIVO, nivolumab; Trach, tracheostomy; IVIG, intravenous immunoglobulin

His muscle strength and laboratory tests improved after the initiation of steroids (Fig. 1). However, on December 12, 2014, he was noted to have increasing use of accessory muscles to assist in breathing. Due to his worsening respiratory status, which was also thought to be secondary to muscle weakness, infliximab was administered as an additional immunosuppressive agent and the patient was also intubated.

On December 12, 2014, a paraneoplastic antibody panel was negative with the exception of elevated anti-SM-titer (1:61440 [normal ≤ 1:120]). The presence of elevated anti-SM-titer provided a potential immunologic mechanism for the patient’s presentation with rhabdomyolysis and polymyositis. We had not previously checked an anti-SM-titer prior to treatment but, we had a pre-treatment blood sample and used it to retrospectively evaluate the patient’s pre-treatment anti-SM-titer. We found the patient’s pre-treatment anti-SM-titer was already elevated at 1:15360. In retrospect, it appears that this patient had an underlying autoimmune disorder that was exacerbated by treatment with nivolumab plus ipilimumab immunotherapy.

**Fig. 2:**
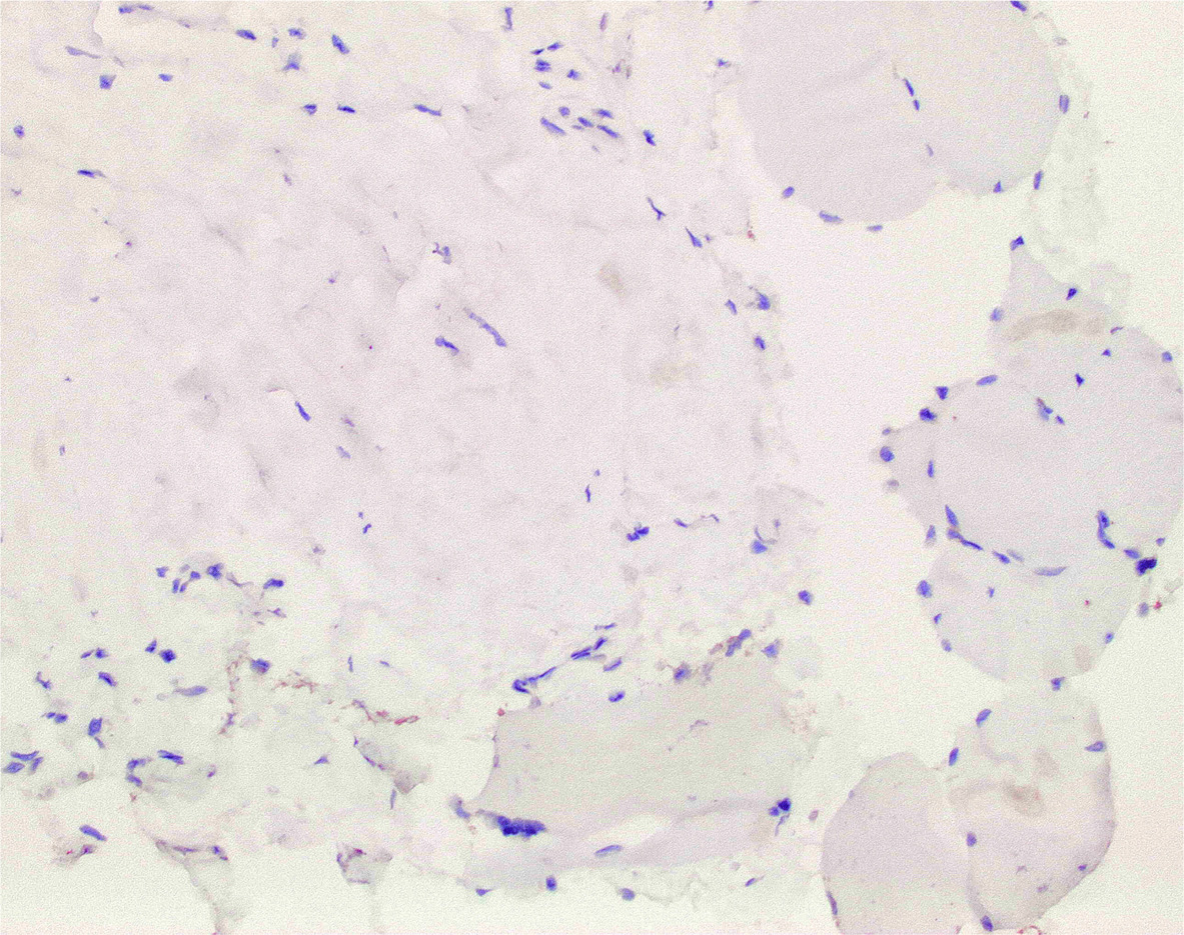
Negative CD3 staining of patient’s muscle biopsy, (20X)

On December 15, 2014, a muscle biopsy of the patient’s left thigh revealed fragments of connective tissue and skeletal muscle fibers with a single minute focus showing a multinucleate giant cell juxtaposed to a muscle fiber with very mild features of degeneration. We analyzed the muscle biopsy, which did not show any immune cell infiltration and this was likely due to the fact that the patient already received immunosuppressive therapies prior to the biopsy (Fig. 2).

The patient ultimately received 2 doses of intravenous immunoglobulin, which was completed in December 2014, and 12 rounds of plasmapheresis, which completed in February 2015. These treatments resulted in improvement of his antibody titer and other laboratory tests (Fig. 1). The patient’s muscle strength improved and tracheostomy was performed in December 2014 to provide intermittent respiratory support while the patient was participating in physical therapy rehabilitation. Unfortunately, on March 2015, imaging studies showed progressive disease and the patient was referred to hospice care where he died in April 2015.

## Discussion

Immune checkpoint therapies have demonstrated high response rates and prolonged overall survival in cancer patients; however, these therapies require education to recognize and treat related toxicities [4, 5, 7–9]. The most common reported grade 3–4 adverse events in combination therapy with ipilimumab plus nivolumab were gastrointestinal, skin and hepatic [10]. Less common reported immune related adverse events include limbic encephalitis, Guillain-Barre syndrome, red cell aplasia, and nephritis.

Polymyositis associated with immune checkpoint therapies has been reported before. Hunter et al. described a patient who developed autoimmune inflammatory myopathy after treatment with ipilimumab [11]. Liao et al. reported atypical neurological complications of ipilimumab, such as chronic inflammatory demyelinating polyneuropathy, transverse myelitis, or concurrent myositis and myasthenia gravis–type syndrome [12]. Yoshioka et al. reported a patient who experienced respiratory discomfort 7 weeks after nivolumab infusion [13]. In another case report, authors reported dermatomyositis 2 weeks after the first dose of ipilimumab therapy [14]. Min et al. reported a patient who was treated with anti-PD1 and experienced severe hypothyroidism and rhabdomyolysis [15]. The cases highlight rhabdomyolysis and polymyositis as notable adverse event with immune checkpoint therapy. An underlying immunologic mechanism for these previous reports of rhabdomyolysis and polymyositis has not been established. Here, we propose that immune checkpoint agents may exacerbate underlying autoimmune conditions consisting of elevated preexisting anti-SM-titer, which can elicit rhabdomyolysis and polymyositis.

## Conclusions

Here, we describe irAEs that may be linked to subclinical autoimmune conditions with elevated levels of pre-existing anti-SM-titer, which highlights the need for additional studies to identify patients who are at risk for toxicities. Future studies will be needed to determine the prevalence of elevated anti-SM-titer cancer patients and to determine whether these patients are at higher risk of developing rhabdomyolysis and polymyositis with immune checkpoint therapies. We believe that our experience may be informative for treating physicians since immune checkpoint therapies are becoming widely used in oncology.

## Abbreviations

AE, adverse event; ALT, alanine transaminase; anti-SM-titer, anti-striated muscle antibody titer; AST, aspartate transaminase; CK, creatine kinase; CK-MB, creatinine kinase isoenzyme MB; CTCAE, Common Terminology Criteria for Adverse Events, Version 4; IPI, ipilimumab; irAEs, immune-related adverse events; IVIG, intravenous immunoglobulin; NIVO, nivolumab; Trach, tracheostomy; ULN, upper limit of normal
